# Automatic Optic Disc Detection in Color Retinal Images by Local Feature Spectrum Analysis

**DOI:** 10.1155/2018/1942582

**Published:** 2018-06-14

**Authors:** Wei Zhou, Hao Wu, Chengdong Wu, Xiaosheng Yu, Yugen Yi

**Affiliations:** ^1^College of Information Science and Engineering, Northeastern University, Shenyang, China; ^2^Engineering Faculty, University of Sydney, NSW 2006, Austria; ^3^Faculty of Robot Science and Engineering, Northeastern University, Shenyang, China; ^4^School of Software, Jiangxi Normal University, Nanchang, China

## Abstract

The optic disc is a key anatomical structure in retinal images. The ability to detect optic discs in retinal images plays an important role in automated screening systems. Inspired by the fact that humans can find optic discs in retinal images by observing some local features, we propose a local feature spectrum analysis (LFSA) that eliminates the influence caused by the variable spatial positions of local features. In LFSA, a dictionary of local features is used to reconstruct new optic disc candidate images, and the utilization frequencies of every atom in the dictionary are considered as a type of “spectrum” that can be used for classification. We also employ the sparse dictionary selection approach to construct a compact and representative dictionary. Unlike previous approaches, LFSA does not require the segmentation of vessels, and its method of considering the varying information in the retinal images is both simple and robust, making it well-suited for automated screening systems. Experimental results on the largest publicly available dataset indicate the effectiveness of our proposed approach.

## 1. Introduction

**Figure 1:**
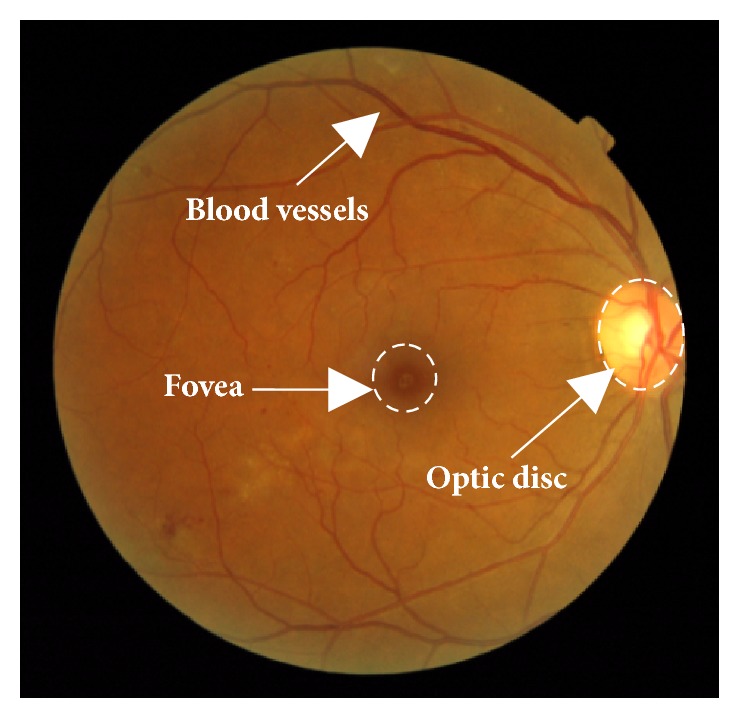
Some anatomical features in retinal fundus images.

Computer based retinal image analysis was first implemented in 1974 [1] and it is now becoming a mainstream technique for quick and accurate detection of retinal diseases such as diabetic retinopathy (DR) and glaucoma [2]. Several important anatomical features appear in the fundus images, such as the retinal blood vessels, the optic disc, and the fovea, as shown in Figure 1. Among these anatomical features, the optic disc appears as a bright, circular-shaped anatomical structure on which the retinal blood vessel network converges (see Figure 1). The position and radius of the optic disc can be used as the references for approximating fovea detection. In addition, the size and shape of the optic disc outer boundaries are useful for diagnosing glaucoma [3]. Therefore, the optic disc is an important anatomical feature in the retinal images, and its detection is a prerequisite for developing automatic screening systems.

Recently, many optic disc detection studies have been presented. Some of them are reviewed below.

The existing works mainly use features such as intensity, appearance, and shape to locate the position of the optic disc. Sinthanayothin et al. [4, 5] proposed optic disc detection methods based on the highest variability in the intensity of adjacent pixels. However, these algorithms do not work well when large areas of bright lesions exist that are similar to the optic disc. Shape is also a useful feature for describing optic discs. Park et al. [6] employed both the round-shape and brightness to detect the optic disc in retinal images. This approach first selects several rounded areas with high intensity variations. Then, the Hough transform is used to estimate the optic disc contour. Finally, the optic disc location is determined by the circle with the highest intensity. However, diseased areas and noisy spots within the retinal images may also present as bright circular lesions that appear similar to the optic disc, which weakens the detection performance. Moreover, optic discs have large variances (e.g., in shape, color, and size), especially in the presence of retinopathies. Hence, localizing the optic disc using only these properties is insufficient [7]. To improve the optic disc detection accuracy, some template matching-based approaches have been proposed to approximately locate the optic disc [8–10]. However, the range of color and contrast variations and the possible presence of pathologies that present as bright spots make it difficult to find a suitable template for matching optic discs. In addition, most of the recently proposed techniques attempt to use information provided by the retinal vasculature to identify the optic disc. In [11], optic disc localization was based on the major blood vessels and their intersection. Hoover et al. [12] identified optic discs using geometrical relationships between the optic disc and blood vessels. Youssif et al. [13] used the vessels' directional matched filter (VDMF) and the outgoing vessels to locate the center of the optic disc. Zhang et al. [14] proposed an optic disc center detection method that used multiscale Gaussian filtering and a VDMF. The optic disc center is determined by the pixel with the smallest difference from its surrounding pixels. The use of a vascular tree can improve the performance of optic disc detection, especially when the optic disc is not clearly visible due to poor contrast or retinal pathologies within the images. However, the accurate segmentation of the vascular tree is a complex and time-consuming task. Apart from the above-mentioned approaches, some machine learning techniques have been incorporated into optic disc localization. Perez et al. [15] utilized a cascade classifier to detect the optic disc. In [15], the cascade classifiers are trained using Haar features extracted from the segmented optic disc and nonoptic disc images. The main drawback of this approach is its computational complexity. Li et al. [16] combined Principle Component Analysis (PCA) with active shape techniques to determine the center of the optic disc by the minimum distance between the original retinal image and its projection onto “disk space”. However, it is difficult to choose a suitable shape model to detect the various disc shapes resulting from many pathological changes.

Although the above-mentioned works have achieved good performances, each method has its own limitations. In summary, the complex vessel structures and its occlusion position variations on the optic disc are two main challenging issues. In other words, we need to detect the optic disc from a complex scene.

In pattern recognition and computer vision, the idea of global frequency with local spatial constraints is widely employed to the complex scene classification [17]. Inspired by it, Li et al. used intermediate representations before classifying scenes [18]. In their approach, they model an image as a collection of local patches. Each patch is obtained from a large vocabulary of codewords. Their goal is to learn a model that best represents the distribution of the codewords of a particular image. Furthermore, it is easier to understand a complex scene by going through a generative model.

**Figure 2:**
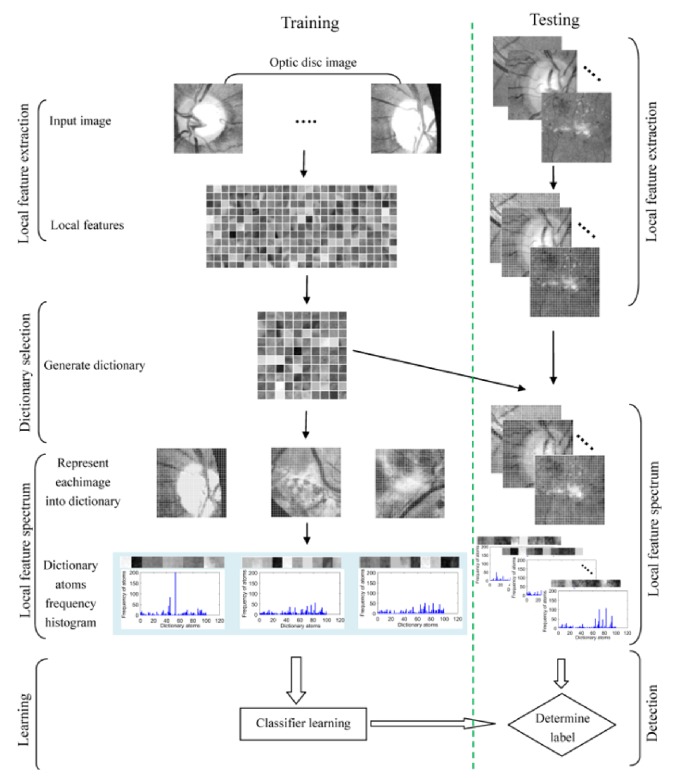
The flow chart of our work.

Optic disc always varies in terms of appearance, size, and location in different retinal images. Humans can recognize images containing optic discs effortlessly by observing parts of the local features of a retinal image, but machines seem to have a hard time doing the same task. Inspired by this, we propose a novel feature extraction method named local feature spectrum analysis (LFSA), which eliminates the influence caused by the variance in the spatial positions of local features. The local feature spectrum is stable for most optic discs. Consequently, we need only a few training samples to achieve a satisfactory detection performance. A flow chart of our approach is depicted in Figure 2.

The remainder of this paper is organized as follows: Section 2 describes the process of candidate extraction.  Section 3 presents the proposed optic disc detection approach. Experimental results are depicted in Section 4 and Section 5 concludes the paper.

## 2. Candidate Extraction

By utilizing some basic image processing techniques, we can acquire a series of candidates and some of them contain the optic disc. And then, the optic disc will be finally identified using the proposed LFSA approach. In this section, the candidate extraction is given briefly.

There are many approaches that have been proposed for candidate extraction such as intensity-based thresholding [4, 5] and template matching [6, 8–10]. Here, we employ our previous study [19] to extract the optic disc candidates which uses a two-step reconstruction based saliency detection approach. It is particularly suitable for those retinal fundus images with uneven illumination and poor contrast. Meanwhile, the optic disc often appears as the area with the highest contrast compared to the background in the red channel [20, 21]. Therefore, the red channel image is employed in this paper.

**Figure 3:**
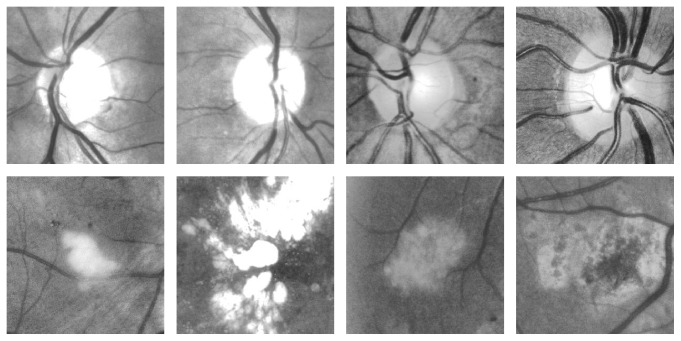
Examples of extracted optic disc regions (the first row) and nonoptic disc regions (the second row).

Some of the optic disc and nonoptic disc candidate example images are shown in Figure 3, in which the first row of images is the extracted optic disc images and the second row of images is the nonoptic disc images.

## 3. The Proposed Method

The proposed optic disc detection approach consists of the following four steps. First, local features are extracted from the retinal fundus images. Second, with the use of the extracted local features, an optimal dictionary is generated. Third, the frequencies of every atom in the dictionary used for new image reconstruction are considered as a kind of “spectrum”. Finally, we use the generated “spectrum” feature for optic disc classification. Each step of the proposed method will be described in detail as follows.

### 3.1. Local Feature Extraction

**Figure 4:**
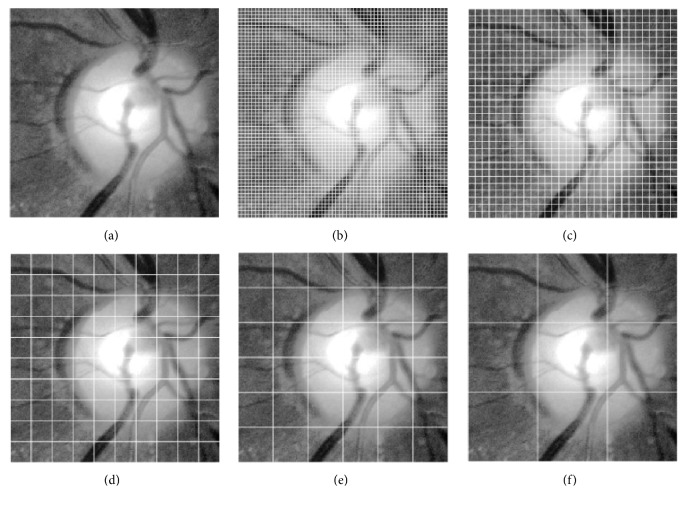
Image partition results using different patch sizes. (a) Original image; (b-e) different patch sizes (from left to right: 5 × 5, 10 × 10, 30 × 30, 50 × 50, and 100 × 100 pixels).

After completing candidate extraction, we obtain a series of subimages (candidates) whose size is 300 × 300 pixels. Then, we define small image patch sizes of *M* × *M* pixels as the local features. Each retinal image is partitioned into* n* nonoverlapping patches where *n* = (300/*M*)^2^. Each patch is ordered lexicographically and denoted as *r*_*i*_ ∈ *ℜ*^*d*×1^(*i* = 1,2, ..., *n*) where *d* = *M* × *M*. Subsequently, the full set of the local features of a retinal image can be denoted as *R* = [*r*_1_, *r*_2_, ..., *r*_*n*_] ∈ *ℜ*^*d*×*n*^. Here, the parameter* M *ranges among the values in the set {5,10, 30, 50,100}. The corresponding segmentation results are depicted in Figure 4.

### 3.2. Dictionary Selection

A dictionary used to reconstruct a whole image can be acquired by using local features. Since the dictionary can directly determine the reconstruction error and the final classification performance, how to select an optimal dictionary is a key issue.

Traditional intermediate representation based models [17, 18] obtain a dictionary of local features by clustering local image descriptors extracted from images. However, clustering methods have several limitations. For example, the number of clusters* k* needs to be specified in advance, which is considered to be one of the greatest drawbacks of these algorithms. Furthermore, the algorithms prefer clusters of approximately similar size, because they always assign an object to the nearest centroid. This often leads to incorrectly defined borders between clusters. Additionally, the clustering process does not consider representational ability; consequently, some clusters will be imported that are useless for image reconstruction tasks. All these limitations affect the classification performance.

In this subsection, with the aim of representing all candidates fully, we employ the sparse dictionary selection approach proposed by Cong et al. [22] to construct an optimal dictionary. Each sample is given a weight that reflects its representational ability. The most representative samples are selected as dictionary atoms [23–28].

After image partitioning, we obtain many equal-sized patches from all the training images. Let *X* = [*x*_1_, *x*_2_, ..., *x*_*N*_] ∈ *ℜ*^*d*×*N*^ denote the data matrix that includes all patches. Here, *x*_*j*_ ∈ *ℜ*^*d*×1^ denotes the* j*-th sample and *N* is the number of samples. Our goal is to select an optimal subset to form a dictionary *X*′ = [*x*_1_′, *x*_2_′, ..., *x*_*M*_′] ∈ *ℜ*^*d*×*Dsize*^ such that the set *X* is well represented by the *X*′ with the smallest *Dsize*. This dictionary selection task can be achieved by solving the following optimization problem:

(1) $$\begin{array}{c} \underset{B}{\min}\frac{1}{2}{\left\|\;X-XB\;\right\|}_{F}^{2}+\lambda {\left\|\;B\;\right\|}_{2,1}, \end{array}$$

where *B* ∈ *ℜ*^*N*×*N*^. Here, ‖*B*‖_*F*_≔∑_*i*,*j*_*B*_*ij*_^2^ is defined as the Frobenius norm of *B* and ‖*B*‖_2,1_≔∑_*i*=1_^*N*^‖*B*_*i*._‖_2_ is defined as the* l*_2,1_-norm of *B*, where *B*_*i*._ denotes the* i*-th row of *B*.

The objective function in (1) consists of two terms, the first term is the representation error, and the second term is the regularization term. The* l*_2,1_-norm is a general version of the* l*_1_-norm when *B* is a vector, i.e., ‖*B*‖_2,1_ = ‖*B*‖_1_. In addition, by constructing a new vector *b* ∈ *ℜ*^*N*^ with *b*_*i*_ = ‖*B*_*i*._‖_2_, ‖*B*‖_2,1_ is equivalent to ‖*b*‖_1_. From this point, it is easy to conclude that minimizing (1) will lead to row sparsity in the representative matrix *B*; i.e., *B* usually contains some zero rows because the corresponding samples in* X* were not selected as dictionary atoms [22]. Here, *λ* is a parameter used to balance the first term and the second term. A larger *λ* leads to more zero rows in *B*.

As shown in (1), the objective function involves the* l*_2,1_-norm, which is nonsmooth and cannot be solved by a closed form [22]. Therefore, in this paper, we adopt the Nesterov [29] algorithm to solve the optimization problem. After obtaining the optimal *B*, we compute each sample's weight using ‖*B*_*i*._‖_2_  (*i* = 1,2, ..., *N*) and sort all the weight values in descending order. Finally, we select the top* Dsize* ranked dictionary atoms to construct the optimal dictionary* V*.

**Figure 5:**
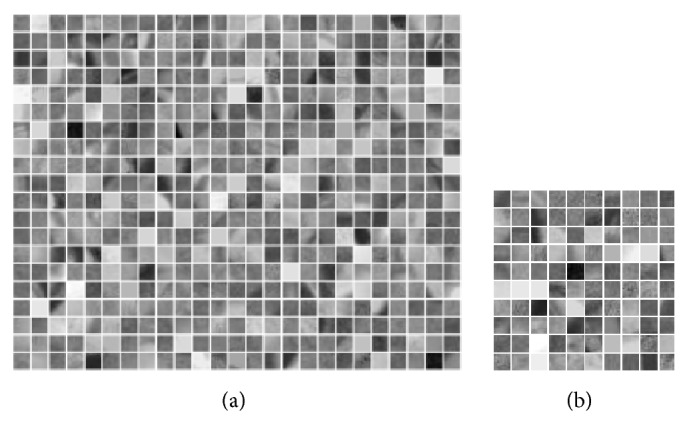
(a) A portion of the example patches used to construct the dictionary; (b) the selected dictionary.

Here, using the patch size parameter* M*=10 and the number of dictionary atoms* Dsize*=100 as an example, the optimal dictionary *V* ∈ *ℜ*^*Dsize*×*N*^ can be obtained by the above-mentioned dictionary selection approach. Figures 5(a) and 5(b) depict the segmented image patches and the selected optimal dictionary, respectively.

### 3.3. Local Feature Spectrum

Based on the obtained dictionary* V*, we can represent an image as a “bag” of “dictionary atoms”, disregarding the atoms' order. For example, for each atom *w* in* V*, the local feature spectrum analysis model estimates the frequencies of atoms in an image as follows:

(2) $$\begin{array}{c} LFSA\left(\;w\;\right)=\overset{n}{\underset{i=1}{\sum }}\left\{\begin{array}{cc} 1 & if\;\;w=\underset{v\in V}{\arg\min\left(D\left(v,r_{i}\right)\right)};\\ 0 & otherwise, \end{array}\right. \end{array}$$

where* n *is the number of local features in an image, *r*_*i*_ is the* i-*th local feature, and *D*(*v*, *r*_*i*_) is the distance between a dictionary atom* v* and a local feature *r*_*i*_. The atom with the smallest distance calculated by *D*(*v*, *r*_*i*_) is used to represent the current patch.

**Figure 6:**
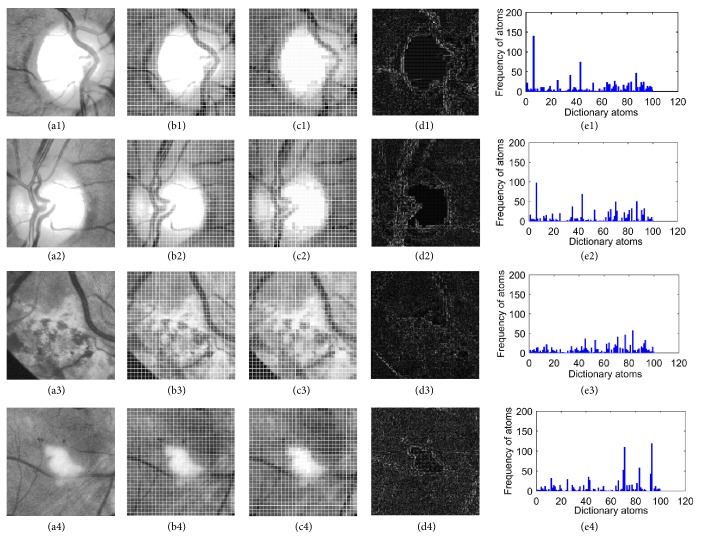
Local feature spectrum analysis. (a1–a4) Original optic disc and nonoptic disc images; (b1–b4) patch segmentation with 10 × 10 pixels; (c1–c4) reconstruction results of original images; (d1–d4) the error images between the original images (a1–a4) and the reconstructed images (c1–c4); (e1–e4) local feature spectrum analysis results for (a1–a4), respectively.

Figure 6 gives the details of local feature spectrum extraction. First, given several candidate region images obtained by our previous approach (see Figures 6(a1)–6(a4)), we partition them into a series of patches, regarded as local features (see Figures 6(b1)–6(b4)). Second, for each patch, the atom with the smallest distance to the current patch is selected to reconstruct it. This process is repeated until all the patches have been reconstructed. Then, we can obtain the full reconstructed images (see Figures 6(c1)–6(c4)) and the corresponding error images, which are calculated by subtracting the original image (see Figures 6(a1)–6(a4)) from the reconstructed image (see Figures 6(c1)–6(c4)) as shown in Figures 6(d1)–6(d4). Finally, we describe an image by the frequency over dictionary atoms as shown in Figures 6(e1)–6(e4).

### 3.4. Classification

In this subsection, we employ the generated local feature spectra (see Figures 6(e1)–6(e4)) as the features for optic disc classification. Two widely used classifiers including* k*-Nearest Neighbor (*k*NN) [30] and the support vector machine (SVM) [31] are utilized in this paper.

First, the obtained local feature spectra of all the candidates can be expressed as a set of* Dsize* dimensional features:

(3) $$\begin{array}{c} F=\left\{\;f_{1},f_{2},\ldots ,f_{i},\ldots ,f_{S}\;\right\}, \end{array}$$

where *f*_*i*_ ∈ *ℜ*^*Dsize*×1^ denotes the* i-*th candidate and* S* is the number of candidates.

Then, each of these features is normalized to a vector with zero mean and unit variance by applying

(4) $$\begin{array}{c} f^{\prime }=\frac{f-\mu }{\sigma }, \end{array}$$

where *μ* and *σ* are the mean and standard deviation vector of the feature, respectively.

Finally, with the use of these normalized spectrum features, we can train the classifier for distinguishing the optic disc candidates from the nonoptic disc candidates, achieving classification.

However, one main problem occurs when performing binary classification for computer-aided diagnosis of medical images. That is, the object candidate (positive samples) features we are trying to identify are similar, whereas the features of nonobject candidates (negative samples) vary widely. For example, in our study, the local feature spectra of optic disc candidates are stable and similar, whereas the local feature spectra of nonoptic disc candidates are varied, as shown in Figure 6. When training a binary classifier, it is desirable that the negative samples should appear in sufficient quantity and species. Therefore, the training set should contain most types of nonobject candidates. Otherwise, the trained model will not achieve a good classification performance. Meanwhile, the number of positive samples should approximately equal the number of negative samples to avoid the class imbalance problem, which also substantially reduces classification accuracy [32]. However, collecting all types of nonobject candidates is difficult. Therefore, we employ a one-class classifier to identify objects of a specific class among all the objects by learning from a training set containing only one-class object.

As an unsupervised machine learning algorithm, one-class SVM (also called support vector data description or support vector domain description (SVDD)) was proposed by Schölkopf et al. [33] to estimate high-dimensional distributions. SVDD first maps the data from the original space to the feature space using a nonlinear transformation and then finds the hypersphere with the minimum volume in the feature space. Parameters such as the kernel parameter and slack factor in SVM and SVDD or the value of* k* in* k*NN are determined using cross validation [34].

## 4. Experimental Results and Analysis

### 4.1. Database

In this section, we employ the publicly available Messidor database to evaluate the proposed approach [35]. The Messidor database contains 1,200 color fundus images with three different resolutions: 1,440 × 960, 2,240 × 1,488, and 2,304 × 1536 pixels. Each image has a reference standard marking every optic disc and agreed upon by the consensus of 4 experts. All the images in Messidor were acquired by three ophthalmologic departments using a color video 3CCD camera on a Topcon TRC NW6 non-mydriatic retinograph with a 45-degree field of view.  Table 1 lists the detailed information of this database. In our experiment, all the retinal images are resized to 1,440 ×960 pixels using bilinear interpolation.

From the Messidor database, after performing candidate extraction, we obtained 1,200 optic disc candidates and 10,985 nonoptic disc candidates. In our experiments, we selected all the optic disc samples and the same number of nonoptic disc samples for training and testing. Classification accuracy is used as the evaluation criterion in Sections 4.2, 4.3, and 4.4. However, we adopt detection accuracy as the evaluation criterion in the last experiment in Section 4.5. From all the selected samples, we randomly selected 70% for training and used the remaining samples for testing. This random sample selection was repeated 10 times, and we report the average classification accuracy as the final result.

### 4.2. Parameter Selection

The proposed method has two parameters: patch size (*M*) and the number of dictionary atoms (*Dsize*), and determining their values appropriately is important in the proposed approach. Here, the patch sizes are set to 5 × 5, 10 × 10, 30 × 30, 50 × 50, and 100 × 100. The number of dictionary atoms varies with different patch sizes. For example, we tuned the value of the *Dsize* parameter by searching the grids {5,10,15,20,25} and {10,30,50,70,90,100} for patches with 5 × 5 and 10 × 10 pixels, respectively. We tuned *Dsize* on the grid {50,150,300,500,700,1000} by searching for patches with 30 × 30, 50 × 50, and 100 × 100 pixels.

We compared three dictionary selection methods, including* K*-means clustering, sparse dictionary selection, and random selection, under different patch sizes and atom numbers. The* k*NN and SVM algorithms are employed for classification, and classification accuracy is adopted as the evaluation criterion. We also report the average reconstruction error, which is defined as the mean value of the mean square error between the original images and the reconstructed images. The reconstruction error can be calculated as follows:

(5) $$\begin{array}{c} R\_error=\frac{1}{T}\overset{T}{\underset{t=1}{\sum }}{\left\|\;I_{t}-I_{t}^{R}\;\right\|}_{2} \end{array}$$

where *I*_*t*_ is the* t*-th original image, *I*_*t*_^*R*^ is the* t*-th reconstructed image, and* T* is the total number of reconstructed images. A smaller error represents a better reconstruction, but the experimental results show that the minimum error does not equate the highest classification accuracy. Therefore, the best number of dictionary atoms will be found via experiment.

Tables 2–6 show the average classification accuracies obtained by two different classifiers (SVM and* k*NN) with three different dictionary selection methods (random selection,* K*-means clustering, and our sparse dictionary selection) and the corresponding reconstruction errors. For example, we denote “SVM + random” as the combination of the SVM classifier with random sample selection dictionary construction for classification, and* R_error* (random) denotes the reconstruction error using a dictionary constructed by random sample selection.

In each table, we fixed the value of* M*. In Table 2, for example, the classification performance obtained by the proposed approach is not good when* Dsize* is small. However, as* Dsize* increases, the classification accuracy improves. The best performance was achieved with* Dsize*=20 using SVM and sparse dictionary selection. Subsequently, the classification accuracy begins to drop, whereas the reconstruction error continues to drop as* Dsize* increases. This result occurs because as the number of dictionary atoms selected for reconstruction becomes larger, the dictionary atoms are more sufficient, leading to a smaller reconstruction error. However, too many atoms introduce noise and cause instability in the local feature spectrum. Thus, although* R_error* always falls as* Dsize* increases, the classification accuracy peaks at a certain* Dsize*.

The five tables correspond to five different values of* M* in the set {5,10,30,50,100}. The best scores corresponding to each* M* are 99% (*Dsize*=20), 99.75% (*Dsize*=90), 98.72% (*Dsize*=150), 96.50% (*Dsize*=300), and 95.01% (*Dsize*=500) using SVM and our dictionary selection approach. Our method's best classification accuracy is 99.75% when* Dsize*=10 and* M*=90. This result demonstrates that the local feature spectrum with* Dsize*=10 and* M*=90 can best reveal the class of an optic disc candidate. Humans tend to find the optic disc by observing local image features with a certain size. When the feature size is too small, all types of images can be represented by the same local features, making the spectra for different classes of candidates inseparable. In contrast, when the feature size is too large, each feature becomes quite specific, which is also unsuitable for reconstructing and recognizing candidate images.

In addition, compared to the dictionary constructed by random selection and* K*-means clustering, the sparse dictionary selection approach performs well for image reconstruction and supports a better classification performance. These results may occur for several reasons. First, building a dictionary by selecting candidates randomly is a simple idea; however, it carries a large risk of including noisy candidates, which affects the reconstruction. Second, in* K*-means clustering, the cluster number is preset and the method does not consider the representative ability of each cluster. Consequently, some useless clusters will be imported for the image classification task. Unlike these two methods, the sparse dictionary selection approach can select the optimal dictionary to reconstruct the candidates. Then, we obtain a dictionary with the minimal number of atoms and discard redundant and noisy samples. Therefore, this approach increases both the accuracy and the computational efficiency.

In all the experimental results, SVM performs better than* k*NN. In particular, when* M*=10 and* Dsize*=90, our approach achieves its best classification performance of 99.75% using “SVM+ours”. In the following experiments, we will regard* M*=10 and* Dsize*=90 as the optimal parameters.

### 4.3. One-Class Classification

**Figure 7:**
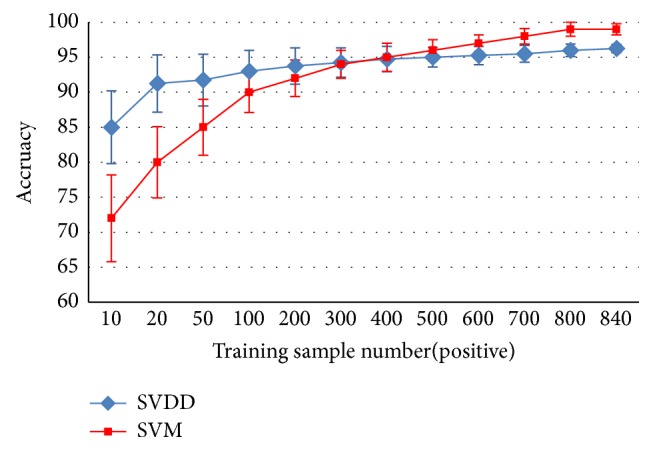
The classification accuracies of SVM and SVDD using different numbers of training samples.

As mentioned in Section 3.4, in practice, the number of nonobjective candidates may be insufficient. Therefore, in this subsection, we compare the performances of an unsupervised one-class SVDD classifier and a supervised binary SVM classifier. The patch size and the number of dictionary atoms are fixed to* M*=10 and* Dsize*=90, respectively. The training samples are randomly selected ten times in the training set, while the testing set remains unchanged. The number of optic disc samples varies from 10 to 840 and the number of nonoptic disc samples is equal to the number of optic disc samples to maintain class balance. As an unsupervised classifier, SVDD does not require nonoptic disc samples. The average classification accuracy is shown in Figure 7. Both the mean value and the standard variance are provided to indicate the classification performances.

As shown in Figure 7, when there are fewer than 400 nonoptic disc samples, SVDD outperforms SVM. For SVDD, the curve tends to become stable after the number of samples exceeds 500, and it reaches its highest accuracy (96%) at 840 samples. For SVM, the curve tends to become stable after the number of samples exceeds 800, and it reaches its highest accuracy (99.75%) after the number of samples exceeds 800. The standard variances of both methods decrease as the number of training samples increase, which means that models obtained using more samples also have more stable prediction and detection performances.

Based on these observations, we can conclude that, on one hand, when only a few positive samples (optic discs) are available, SVDD performs better than SVM. On the other hand, when sufficient negative samples (nonoptic discs) exist, the SVM model is more accurate and stable. In practice, when negative samples are hard to obtain, we should choose the unsupervised one-class classifier SVDD. Alternatively, if higher accuracy is required, we need to acquire enough and sufficient training samples and the supervised binary classifier SVM. For this paper, we can acquire sufficient labeled samples. Therefore, we compare the detection results using SVM with those of other state-of-the-art methods.

### 4.4. Comparison with the Baseline Approach

Here, we regard the classification based on the original gray feature as the baseline approach. That is, we directly utilize the candidates obtained by Section 2 as the input samples for training and testing. The corresponding classification results with different classifiers are listed in Table 7. As Table 7 shows, our approach achieves better performances than the baseline algorithm using both classifiers, which indicates that the local feature spectrum analysis process not only reduces the number of input features but also improves classifier performance.

### 4.5. Comparison with State-of-the-Art Approaches

In the last experiment, to compare the proposed approach with other state-of-the-art approaches, we adopt the accuracy of optic disc detection as the evaluation criterion [30–32]. When the detected optic disc center is within the circumference of the optic disc in the reference standard given in the database, then the detection is considered to be successful.  Table 8 shows the comparison results on the Messidor dataset.

The comparative results listed in Table 8 show that the proposed approach is more reliable than other tested approaches in terms of detection accuracy, indicating the effectiveness of the proposed approach. Specially, traditional approaches [36, 37] including our approach outperform the deep neural network (DNNs) based approach [32]. The main reason is that there is still an improvement room for the novel techniques, especially for small datasets.

**Figure 8:**
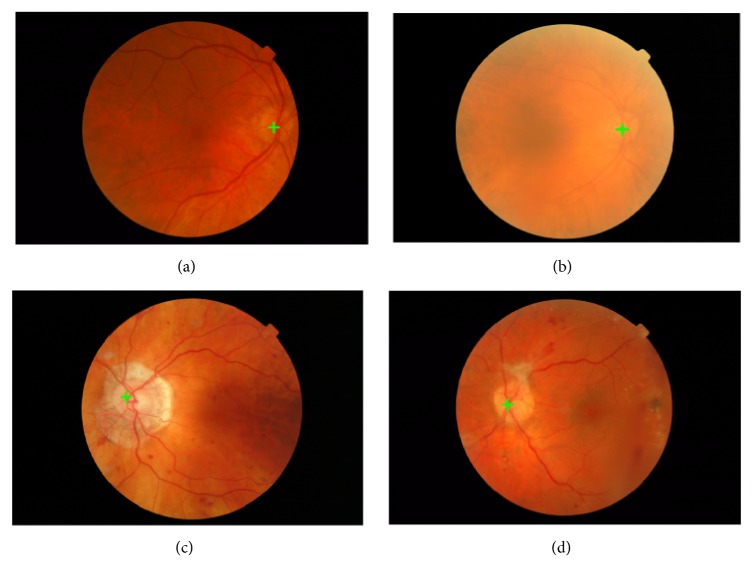
Results of the proposed method. The green cross represents the detected optic disc center. (a) Uneven optic disc brightness with blood vessels; (b) blurred optic disc appearance; (c) optic disc appearance with peripapillary atrophy; (d) myelinated nerve fibers connected to optic disc margin.

Figure 8 shows some examples where the proposed approach successfully detected the optic disc center. The center of the candidate subimage is considered as the center of optic disc and marked with green cross. As shown by Figure 8, the proposed approach is robust to the influence caused by the variations in the optic disc's appearance, size, or location (see Figures 8(a), 8(c), and 8(d)). And it is also robust to uneven illumination, which results in the optic disc appearing indistinct and blurred (see Figure 8(b)). Overall, our approach uses only the local feature spectrum without requiring any other information, such as blood vessels or predesigned templates. Therefore, the proposed approach is both simple and efficient for optic disc detection, especially in abnormal retinal images.

**Figure 9:**
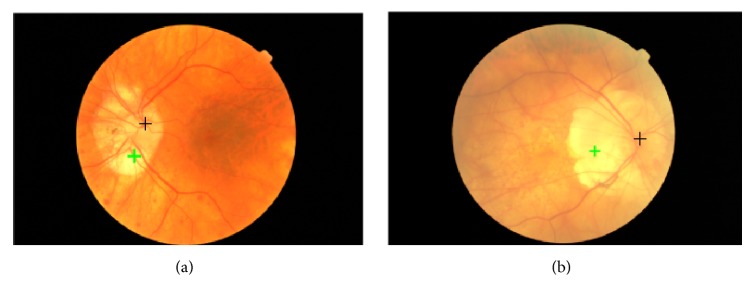
Optic disc detection failure examples. (a-b) Extra-large and bright lesions overshadow the optic disc, making it invisible.

The Messidor database includes two images in which our proposed approach failed to locate the optic disc, as shown in Figure 9. The detected locations of the proposed approach are marked by green crosses, while the ground truth locations are marked by black crosses. In these cases, extra-large and bright lesions overshadow the optic discs, making them invisible. The disc areas are not included in the intensity image (see Figures 9(a) and 9(b)) which should be considered in future work.

## 5. Conclusions

Complex structures and their spatial variability reduce the recognition rate of optic discs. Inspired by the fact that humans can find optic discs in images by observing local features, we propose using local feature spectrum analysis to eliminate the influence caused by the variability of spatial locations. In the proposed approach, each candidate is reconstructed using a set of local features, and the frequencies of all the local features are considered as a type of “spectrum” suitable for the recognition tasks. To find an optimal set of local features, we employ the sparse dictionary selection approach, which aims to find the most representative atoms. We also discuss the common class imbalance problem in medical dataset. When there are insufficient numbers of negative samples, a one-class classifier performs better, whereas when sufficient samples are available, a binary classifier achieves more precise classification performances. The proposed approach is tested on a publicly available Messidor database. The experimental results indicate that the proposed approach yields a better performance than other state-of-the-art approaches.

## Figures and Tables

**Table 1:** Descriptive statistics of the Messidor database.

	Resolution	Coverage of the retina	Number of images
Type	1440 × 960	45°	151
Type	2240 × 1488	45°	881
Type	2304 × 1536	45°	168

**Table 2:** The average classification accuracy rates (%), standard deviations, (%) and reconstruction errors (*R_error*) of the proposed approach with *M*=5.

*Dsize*	5	10	15	20	25
SVM + random	92.01 ±2.15	94.23 ±2.47	95.57 ±2.28	95.04 ±2.63	94.80 ±2.89
SVM + *K*-means	93.67 ±2.44	94.88 ±3.01	96.23 ±2.50	97.67 ±2.37	96.35 ±2.89
SVM + ours	96.25 ±2.42	98.00 ±1.96	98.50 ±1.82	99.00 ±2.29	98.80 ±2.33
*k*NN + random	91.36 ±2.17	92.75 ±2.46	94.37 ±2.86	94.50 ±2.05	94.21 ±2.49
*k*NN + *K*-means	92.69 ±3.26	93.97 ±2.87	95.61 ±2.54	95.11 ±2.63	94.93 ±2.37
*k*NN + ours	94.50 ±2.79	97.25 ±2.33	98.00 ±2.13	98.50 ±2.35	98.31 ±2.64
*R_error* (random)	0.427	0.410	0.407	0.398	0.378
*R_error* (*K*-means)	0.329	0.318	0.312	0.301	0.296
*R_error* (ours)	0.118	0.116	0.108	0.105	0.094

**Table 3:** The average classification accuracy rates (%), standard deviations (%), and reconstruction errors (*R_error*) of the proposed approach with *M*=10.

*Dsize*	10	30	50	70	90	100
SVM + random	94.07 ±2.96	94.79 ±2.78	95.14 ±2.70	96.88 ±2.87	96.62 ±2.34	96.51 ±3.06
SVM + *K*-means	95.63 ±2.45	95.77 ±2.62	96.21 ±2.33	97.96 ±2.91	98.71 ±1.99	97.60 ±2.98
SVM + ours	98.50 ±2.10	98.75 ±1.32	99.20 ±1.93	99.50 ±2.06	99.75 ±1.79	99.65 ±1.96
*k*NN + random	93.89 ±3.12	94.20 ±2.09	95.53 ±2.96	96.34 ±1.98	96.55 ±2.37	96.43 ±2.55
*k*NN + *K*-means	94.22 ±2.93	95.23 ±2.68	96.60 ±2.45	97.59 ±2.85	98.21 ±2.14	97.83 ±2.22
*k*NN + ours	97.25 ±2.13	97.25 ±2.27	98.50 ±2.35	98.64 ±2.13	98.80 ±2.25	98.58 ±2.38
*R_error* (random)	0.449	0.437	0.423	0.415	0.408	0.396
*R_error* (*K*-means)	0.334	0.326	0.320	0.316	0.306	0.297
*R_error* (ours)	0.181	0.176	0.162	0.153	0.140	0.138

**Table 4:** The average classification accuracy rates (%), standard deviations (%), and reconstruction errors (*R_error*) of the proposed approach with *M*=30.

*Dsize*	50	150	300	500	700	1000
SVM + random	92.95 ±3.64	95.79 ±3.86	95.09 ±2.14	94.36 ±2.75	94.17 ±2.49	94.02 ±2.16
SVM + *K*-means	93.83 ±3.33	97.02 ±3.64	97.36 ±2.88	96.24 ±2.69	96.03 ±2.72	95.83 ±2.99
SVM + ours	97.75 ±3.10	98.72 ±3.29	97.75 ±2.67	97.00 ±2.71	96.50 ±2.84	96.50 ±2.76
*k*NN + random	92.14 ±3.69	96.86 ±3.91	93.04 ±2.80	92.96 ±3.87	92.36 ±3.60	90.05 ±3.49
*k*NN + *K*-means	92.96 ±3.59	97.50 ±3.55	93.76 ±2.73	93.45 ±3.93	92.61 ±3.47	91.34 ±3.91
*k*NN + ours	96.50 ±3.17	98.00 ±3.06	94.00 ±2.89	93.75 ±3.04	92.75 ±3.21	91.75 ±3.09
*R_error* (random)	0.463	0.448	0.440	0.436	0.429	0.418
*R_error* (*K*-means)	0.356	0.349	0.336	0.328	0.319	0.311
*R_error* (ours)	0.183	0.178	0.174	0.169	0.168	0.162

**Table 5:** The average classification accuracy rates (%), standard deviations (%), and reconstruction errors (*R_error*) of the proposed approach with *M*=50.

*Dsize*	50	150	300	500	700	1000
SVM + random	91.56 ±2.98	94.76 ±3.77	93.49 ±3.16	94.27 ±2.75	93.17 ±3.73	93.01 ±2.39
SVM + *K*-means	92.98 ±2.75	95.32 ±3.39	94.76 ±3.64	95.19 ±2.63	94.24 ±3.85	93.94 ±2.73
SVM + ours	94.75 ±2.53	95.48 ±3.46	96.50 ±3.10	95.45 ±2.12	94.42 ±3.44	94.40 ±2.53
*k*NN + random	90.13 ±3.17	91.83 ±3.94	94.15 ±3.19	91.37 ±2.34	91.01 ±3.73	90.07 ±2.39
*k*NN + *K*-means	90.87 ±3.61	92.09 ±3.46	95.09 ±3.90	91.88 ±2.65	90.35 ±3.94	90.78 ±2.31
*k*NN + ours	91.95 ±3.11	92.25 ±3.77	96.25 ±3.56	92.00 ±2.89	91.75 ±3.13	91.25 ±2.71
*R_error* (random)	0.472	0.465	0.459	0.447	0.436	0.429
*R_error* (*K*-means)	0.375	0.367	0.358	0.344	0.329	0.320
*R_error* (ours)	0.194	0.187	0.184	0.179	0.177	0.174

**Table 6:** The average classification accuracy rates (%), standard deviations (%), and reconstruction errors (*R_error*) of the proposed approach with *M*=100.

*Dsize*	50	150	300	500	700	1000
SVM + random	91.51 ±3.76	93.16 ±3.49	93.68 ±3.49	94.03 ±3.18	93.11 ±3.71	91.85 ±2.54
SVM + *K*-means	92.56 ±3.61	94.03 ±3.55	94.36 ±3.72	94.87 ±3.54	93.96 ±3.34	92.03 ±2.72
SVM + ours	94.15 ±3.15	94.25 ±3.19	94.75 ±3.47	95.01 ±3.06	94.00 ±3.35	92.25 ±2.94
*k*NN + random	90.07 ±3.89	90.83 ±3.36	92.07 ±3.82	91.36 ±2.44	90.36 ±3.79	89.06 ±3.99
*k*NN + *K*-means	91.11 ±3.13	91.09 ±3.21	92.31 ±3.64	92.89 ±2.30	90.87 ±3.81	89.47 ±3.74
*k*NN + ours	92.50 ±3.34	91.30 ±3.51	92.50 ±3.04	93.23 ±2.98	91.00 ±3.33	89.75 ±3.36
*R_error* (random)	0.486	0.479	0.468	0.456	0.439	0.435
*R_error* (*K*-means)	0.384	0.380	0.371	0.362	0.358	0.349
*R_error* (ours)	0.225	0.217	0.213	0.209	0.207	0.205

**Table 7:** The average classification accuracy rate (%) of the baseline method and our approach using different classifiers.

Classifier	Baseline	Our approach
SVM	93.50	99.75
*k*NN	91.50	98.80

**Table 8:** Successful detection rate (%) of different methods for optic disc detection on the Messidor dataset.

Methods	Successful detection rate (%)
Ahmed et al., [36]	97.80
Aquino et al., [37]	98.83
Al-Bander et al., [38]	97.00
Ours	99.83

## Data Availability

The data used to support the findings of this study are available from the corresponding author upon request.
